# Supplementary material for: Major Adverse Cardiovascular Events and Mortality Prediction by Circulating GDF-15 in Patients with Type 2 Diabetes: A Systematic Review and Meta-Analysis
Source: Biomolecules. 2022 Jul 4;12(7):934. doi: 10.3390/biom12070934 (PMC9312922; doi:10.3390/biom12070934)
Supplement: Supplementary file 1 [file biomolecules-12-00934-s001.zip › biomolecules-1778969-supplementary.pdf]

biomolecules

Supplementary Materials

Table S1. Format and details of source data from the 8 studies included in the meta-analysis.

Study (year)	Original unit of estimated HR for GDF-15 concentration	Expression of average GDF-15 levels	Measurement of dispersion of GDF-15 levels (pg/ml)	GDF-15 quantification assay	Ref.
Carlsson et al. (2020)	Per SD of log_2(GDF-15)	N/A	N/A	Multiplex CVD I 96x96 kit (Olink Proseek)	[14]
Cavusoglu et al. (2015)	Per SD of log(GDF-15)	Median (25th, 75th)	PLTP <17.44: 1134 (855, 1557)<br>PLTP ≥17.44: 1365 (961, 2050)	ELISA (R&D Systems)	[24]
Frimodt-Møller et al. (2018)	Per SD of log_2(GDF-15)	Median (IQR)	1533 (1132–2236)	Elecsys 2010 (Roche)	[13]
Gerstein et al. (2015)	Per SD of log(GDF-15)	N/A	N/A	Human DiscoveryMAP (Rules-Based Medicine)	[25]
Pavo et al. (2016)	Per IQR of log(GDF-15)	Median (IQR)	1391 (881–2176)	ELISA (Roche)	[26]
Resl et al. (2016)	Per log(GDF-15)	Median (IQR)	1474 (906–2178)	ELISA (Roche)	[27]
Sen et al. (2021)	Per doubling log(GDF-15)	Median (IQR)	1774 (1242–2514)	Elecsys (Roche)	[20]
Sharma et al. (2020)	Per log_2(GDF-15)	Median	1246	ELISA (Roche)	[28]

ELISA, enzyme-linked immunosorbent assay; GDF-15, growth differentiation factor-15; HR, hazard ratio; IQR, interquartile range; 3log, logarithmic transformations; N/A, not applicable or available; P, plasma; PLTP, phospholipid protein; S, serum; SD, standard deviation.

Table S2. Summary table of included studies with harmonized hazard ratios and the harmonization methodology used (see text for details)

Study (year)	Original unit of estimated HR for GDF-15 concentration	Expression of average GDF-15 levels	Original estimate of CVD outcomes*	Harmonized estimate of CVD outcomes*	Original estimate of all-cause mortality*	Harmonized estimate of all-cause mortality*	Harmonization methodology	[Ref.]
Carlsson et al. (2020)	Per SD of log_2(GDF-15)	N/A	1.34 (0.96–1.87)	1.34 (0.97–1.86)	N/A	N/A	N/A	[14]
Cavusoglu et al. (2015)	Per SD of log(GDF-15)	Median (25th, 75th)	N/A	N/A	1.91 (1.47–2.49)	1.57 (1.31–1.88)	Eqn. (1)	[24]
Frimodt-Møller et al. (2018)	Per SD of log_2(GDF-15)	Median (IQR)	1.25 (0.85–1.84)	1.25 (0.85–1.84)	1.90 (1.24–2.89)	1.90 (1.24–2.91)	N/A	[13]
Gerstein et al. (2015)	Per SD of log(GDF-15)	N/A	1.18 (1.12–1.24)	1.12 (1.08–1.16)	1.29 (1.20–1.38)	1.19 (1.13–1.25)	Eqn. (1)	[25]
Pavo et al. (2016)	Per IQR of log(GDF-15)	Median (IQR)	2.23 (1.79–2.78)	1.74 (1.50–2.03)	3.55 (2.60–4.84)	2.41 (1.94–2.99)	Eqn. (1), (2) & (3)	[26]
Resl et al. (2016)	Per log(GDF-15)	Median (IQR)	1.37 (1.12–1.68)	1.24 (1.08–1.43)	N/A	N/A	Eqn. (1), (2) & (3)	[27]
Sen et al. (2021)	Per doubling log(GDF-15)	Median (IQR)	1.20 (1.04–1.38)	1.05 (1.01–1.09)	1.32 (1.14–1.54)	1.08 (1.03–1.12)	Eqn. (1), (2) & (3)	[20]
Sharma et al. (2020)	Per log_2(GDF-15)	Median	1.15 (1.04–1.27)	1.13 (1.03–1.23)	N/A	N/A	Eqn. (2) & (3)	[28]

Eqn., equation; HR, hazard ratio; IQR, interquartile range; log, logarithmic transformations; N/A, not applicable/available, ref., reference number, SD, standard deviation.
*effect size estimates were expressed in adjusted hazard ratios (95% confident interval).

Table S3. Assessment of quality of studies using the Newcastle-Ottawa scale.

**Components**		Carlsson et al. [14]	Cavusoglu et al.[24]	Frimodt-Møller et al. [13]	Gerstein et al. [25]	Pavo et al. [26]	Resl et al. [27]	Sen et al. [20]	Sharma et al. [28]
		**Study [Ref. no.]**							
**Selection**	Representative of the exposed cohort	★	★	★	★	★	★	★	★
	Selection of external cohort	★	★	–	–	–	–	★	★
	Ascertainment of exposure [a]	★	★	★	★	★	★	★	★
	Outcome of interest not present at the start of the study [b]	★	–	★	★	★	★	★	★
**Comparability**	Comparability of cohorts [c]: main factor	★	★	★	★	–	★	★	★
	Comparability of cohorts [d]: additional factor	★	★	★	★	–	★	★	★
**Outcome**	Assessment of outcome	★	★	★	★	★	★	★	★
	Sufficient follow-up time [e]	–	★	★	★	★	★	★	★
	Adequacy of follow-up	★	★	★	★	★	★	★	★
**Total score (out of 9)**		8	8	8	8	8	6	8	9

A total score of ≥6 points indicates good quality.
[a] One star was assigned if details about measurement of blood GDF-15 concentration were provided.
[b] One star was assigned if a CVD outcome(s) of interest was not available at the start of study.
[c] One star was assigned for a study that controlled for age.
[d] One star was assigned to a study that controlled for additional factors apart from age.
[e] One star was assigned for mean or median follow-up duration of ≥5 years. –, not applicable

biomolecules

MDPI

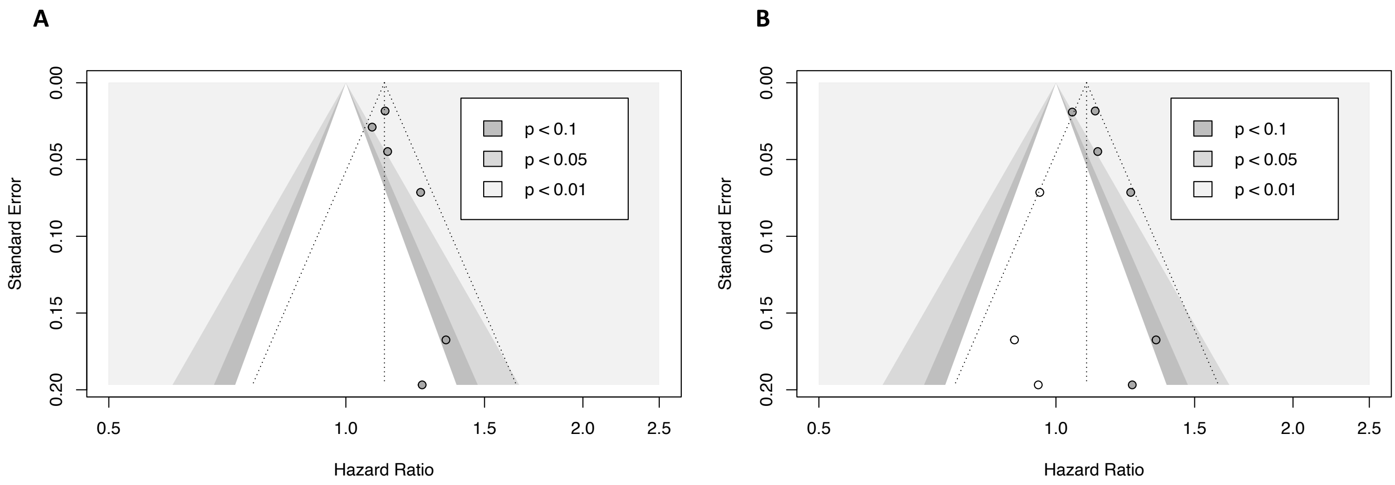

Figure S1. Funnel plots assessing publication bias in the meta-analysis of A) MACE. Effect corrected by small-study bias by Duval and Tweedie's trim and fill test B) shared similar size as pooled effect size A).
